# The Wnt receptor Ryk is a negative regulator of mammalian dendrite morphogenesis

**DOI:** 10.1038/s41598-017-06140-z

**Published:** 2017-07-20

**Authors:** Vanessa Lanoue, Michael Langford, Amanda White, Kai Sempert, Lily Fogg, Helen M. Cooper

**Affiliations:** The University of Queensland, Queensland Brain Institute, Brisbane, Queensland 4072 Australia

## Abstract

The unique dendritic architecture of a given neuronal subtype determines its synaptic connectivity and ability to integrate into functional neuronal networks. It is now clear that abnormal dendritic structure is associated with neuropsychiatric and neurodegenerative disorders. Currently, however, the nature of the extrinsic factors that limit dendritic growth and branching within predetermined boundaries in the mammalian brain is poorly understood. Here we identify the Wnt receptor Ryk as a novel negative regulator of dendritic arborisation. We demonstrate that loss of Ryk in mouse hippocampal and cortical neurons promotes excessive dendrite growth and branching *in vitro*. Conversely, overexpression of wildtype Ryk restricts these processes, confirming that Ryk acts to restrain dendrite arborisation. Furthermore, we identify a hitherto uncharacterized membrane proximal subdomain crucial for Ryk-mediated suppression of dendrite morphogenesis, suggesting that it may act through a novel signalling pathway to constrain dendrite complexity. We also demonstrate that Ryk performs a similar function *in vivo* as *Ryk* haploinsufficient postnatal animals exhibit excessive dendrite growth and branching in layer 2/3 pyramidal neurons of the somatosensory cortex. These findings reveal an essential role for Ryk in regulating dendrite complexity and raise the intriguing possibility that it may influence neural plasticity by modifying dendritic structure.

## Introduction

The unique three-dimensional architecture of the dendritic arbour is fundamental to a neuron’s ability to integrate into functional neuronal networks and dictates the extent and quality of information flow across the network. Dendritic branch length and the degree of branching directly determine spine density and the size of the target field, thereby constraining the number of synaptic inputs^1–3^. Abnormal dendritic arborisation and spine density lead to diminished synaptic connectivity and impaired cognitive function associated with neurodevelopmental conditions, including autism and schizophrenia, as well as neurodegenerative disorders such as Alzheimer’s disease^1, 4, 5^. Therefore, delineating the molecular signalling cascades that govern the elaboration of stereotypic dendritic arbours will not only provide key insights into the fundamental principles guiding the establishment of complex neural circuits, but will also shed light on aberrant processes that contribute to neuropsychiatric conditions and dementia.

During the developmental phase, dendritic morphology is highly dynamic due to continual extension and retraction of branches which are stabilized by neuronal activity, thereby promoting synapse maturation^1, 6–8^. Members of the Wnt family of morphogens and their receptors have been identified as positive regulators of dendrite growth and branching, and act through several distinct but interconnected pathways that primarily target the actin and microtubule cytoskeletons^1, 9–11^. Wnt7b increases dendritic branching in an activity-independent manner via the Dishevelled/Rac/JNK pathway (Planar Cell Polarity (PCP) pathway), a major actin remodelling pathway^11^. Wnt5a/Fzd4 interactions promote dendrite branching and growth via the downstream effectors PSD95 and GRIP1 (glutamate receptor interacting protein 1)^12^. Activity-dependent induction of Wnts is also a major contributor to dendritic arborization^13, 14^. In this context, Wnts increase dendritic complexity through a β-catenin/N-cadherin/actin pathway^14^. A recent study has linked mutations in *FZD9* to the increased dendrite growth and spine density observed in William’s syndrome^15^, demonstrating that Wnt signalling can also limit dendritic complexity. However, the Wnt signalling pathway responsible for this inhibitory activity has not yet been identified.

The Wnt receptor Ryk was first identified as an axon guidance receptor that mediates Wnt5a repulsion, and is essential for the guidance of layer 2/3 pyramidal axons across the corpus callosum and layer 5 projections along the corticospinal tract^16–20^. More diverse roles have since emerged for Ryk in a range of neurodevelopmental processes, including neural tube formation, neuronal differentiation in the early cortex, the induction of GABAergic interneuron fate and the suppression of oligodendrocyte identity in the ventral forebrain^21–23^. Ryk is known to use the three principal Wnt pathways^9, 24, 25^, depending on the biological context. Wnt5a/Ryk mediate axon outgrowth and chemorepulsion via the Wnt/Ca^2+^ pathway^17, 19^, whereas in the neural tube, Ryk establishes neuroepithelial polarity through the PCP pathway^23^. In *C. elegans* Ryk activates β-catenin-dependent Wnt signalling during vulva cell fate specification^26^.

Recent studies in *Drosophila* have uncovered paradoxical functions for Ryk in dendrite morphogenesis. Wnt5a/Ryk interactions confine dendritic territories on the adult *Drosophila* epidermis by restricting dendrite growth^27^. In this context, Ryk promotes Wnt5a signal transduction. In contrast, Ryk inhibits Wnt5a-mediated dendrite repulsion in the developing *Drosophila* antennal lobe^28^. A recent study has provided preliminary evidence that Ryk may be involved in the initiation of dendrite sprouting in the mouse^29^. However, to date, the involvement of Ryk in dendritic growth and arborisation has not been explored in the mammalian system. Here we demonstrate that Ryk is a negative regulator of dendrite arborisation in mouse cultured hippocampal and cortical neurons and provide evidence that it acts through an alternative pathway to restrict dendrite complexity. We further demonstrate that Ryk functions *in vivo* to constrain dendritic complexity in layer 2/3 pyramidal neurons of the postnatal somatosensory cortex.

## Results

### *Ryk* is expressed in neurons undergoing dendritic arborisation

**Figure 1:**
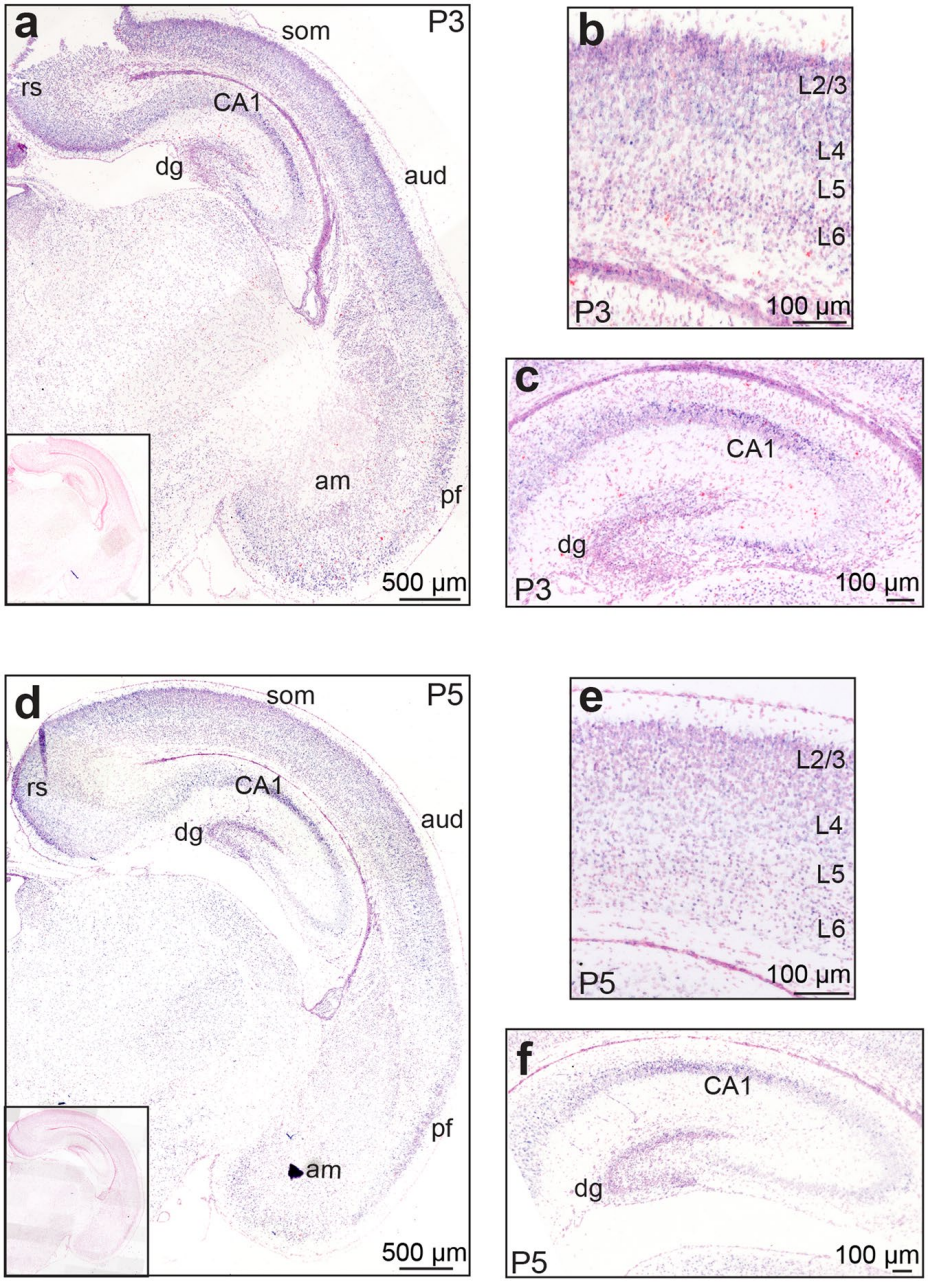
*Ryk* is expressed in the neocortex and hippocampus during dendritogenesis. *In situ* hybridization at P3 (**a**,**b**,**c**) showed that *Ryk* mRNA was present throughout the cortex where it was most intensely expressed in layer 2/3 (L2/3) of the somatosensory cortex (som) (**a**,**b**). Strong expression was also seen in the granule cells of the dentate gyrus (dg) and in the pyramidal cells of the CA1 region of the hippocampus (**a**,**c**). *Ryk* expression remained high in these areas at P5 (**d**,**e**,**f**). (**a**,**d**) Insets: no hybridization signal was seen with control riboprobes. am, amygdala; aud, auditory cortex; pf, piriform cortex; rs, retrosplenial cortex.

In early postnatal life dendrites vigorously extend and retract processes prior to stabilization following synapse formation^1, 3, 6^. Before extensive dendritogenesis occurs (embryonic day 18.5, E18.5) *Ryk* mRNA is present at moderate levels in layer 2/3 of the mouse cortex and at lower levels in layers 4, 5 and 6, whereas it is less intensely expressed in the hippocampus (Allen Brain Atlas, http://www.brain-map.org). To determine the *Ryk* mRNA expression pattern in the postnatal brain we performed *in situ* hybridization analysis which revealed that *Ryk* was expressed broadly throughout the neocortex at postnatal days 3 and 5 (P3, P5) (Fig. 1). The highest level of *Ryk* mRNA was seen in layers 2/3 of the somatosensory and auditory cortices, whereas lower expression was seen in neuronal populations within layers 4, 5 and 6 (Fig. 1a,b,d,e). Strong *Ryk* expression was also observed in the retrosplenial and piriform cortices and the amygdala (Fig. 1a,d). At P3 and P5 the granule cells of both the dentate gyrus and the pyramidal neurons within the CA1 region of the hippocampus also exhibited strong *Ryk* expression (Fig. 1a,c,d,f). By P14 *Ryk* levels are greatly decreased in all cortical areas but remain high in the dentate gyrus and CA1 region (Allen Brain Atlas, http://www.brain-map.org). Therefore *Ryk* is most intensely expressed in cortical and hippocampal neurons as they undergo dynamic dendritic growth and arborisation, suggesting that it may play an important role in dendrite morphogenesis.

### Ryk suppresses dendrite growth and branching in hippocampal neurons

**Figure 2:**
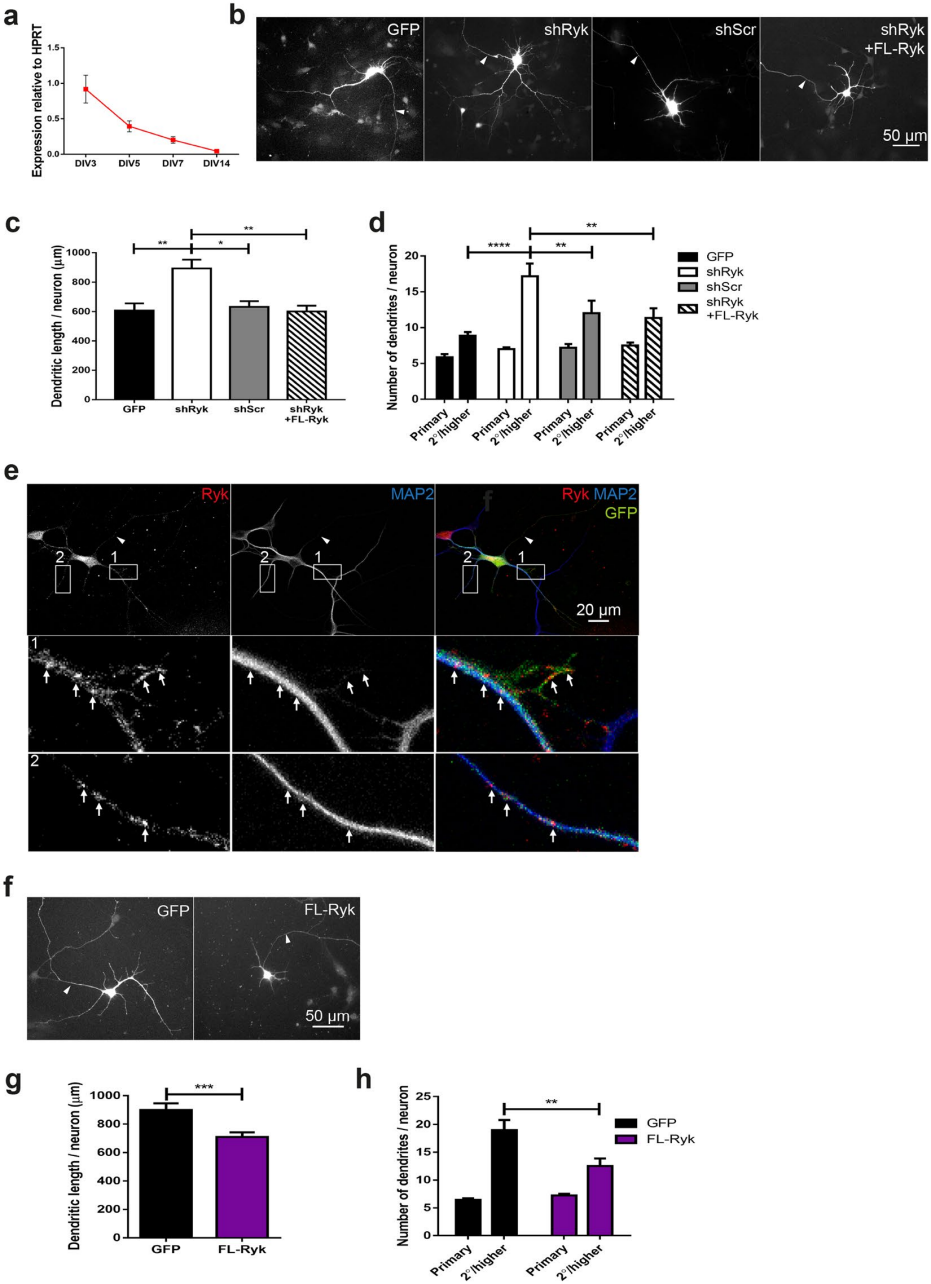
Ryk inhibits dendritic growth and branching in cultured hippocampal neurons. (**a**) Quantitative PCR showed that *Ryk* was expressed in cultured hippocampal neurons. HPRT, hypoxanthine guanine phosphoribosyl transferase. (**b**,**c**,**d**) Plasmids containing Ryk-specific shRNA (shRyk) or control shRNA (shScr) and GFP were transfected into DIV3 hippocampal neurons and dendritogenesis assessed at DIV5. Depletion of Ryk resulted in an increase in dendritic length (**b**,**c**) and the number of secondary and higher order (2°/higher) dendrites (**b**,**d**). Coexpression of shRNA-resistant FL-Ryk with shRyk fully rescued dendritic growth and branching (n = 6; 50–60 neurons/condition; *p < 0.05; **p < 0.01; ****p < 0.0001). (**e**) When transfected alone, FL-Ryk, identified by an anti-Myc antibody, localized to the dendritic shaft and filopodia of hippocampal neurons. Dendrites were identified using anti-MAP2. Bottom panels 1 and 2 are enlargements of insets. Arrows point to FL-Ryk clusters. Overexpression of FL-Ryk alone in hippocampal neurons led to a decrease in dendritic length (**f**,**g**) and 2°/higher order branching (**f**,**h**) (n = 6; 50–60 neurons/condition; **p = 0.0015; ***p = 0.0008). Data are represented as the mean ± s.e.m. (**b**,**e**,**f**) Arrowheads indicate the axon.

In our previous study we provided preliminary evidence that Ryk controlled dendrite sprouting of cultured cortical neurons at 2 days *in vitro* (DIV2)^29^. Here we test the hypothesis that Ryk plays a key role in the establishment of dendritic structure. We first depleted Ryk in cultured hippocampal neurons from E18.5 C57Bl/6 mouse embryos using RNA interference. Quantitative PCR confirmed the expression of *Ryk* mRNA in cultured neurons during the period of vigorous dendrite expansion (P3–P5) (Fig. 2a). As *Ryk* is most strongly expressed in the granule cells of the dentate gyrus and in the CA1 hippocampal region between P3 and P5 (Fig. 1) we focused our analysis on Ctip2-positive neurons which are restricted to these regions^30^. Ryk knockdown was achieved using a short hairpin RNA (shRyk, control: shScr) or long hairpin miRNA^31^ (miRyk, control: miCo) cloned into vectors encoding GFP. Knockdown efficiency was assessed by cotransfection with full-length mouse Ryk (FL-Ryk) in HEK293T cells. shRyk or miRyk expression resulted in 83% or 84% reduction of Ryk, respectively (Supplementary Fig. 1a,b).

Interfering RNAs were transfected into hippocampal neurons at DIV3 and the number of branches and dendrite length were quantified 48 h later using the NeuronJ plugin of ImageJ. shRNA depletion of Ryk resulted in a significant increase in total dendrite length per neuron compared to GFP alone or control cells expressing shScr (Fig. 2b,c). This effect was rescued by cotransfecting shRNA-resistant mouse Ryk, confirming that this phenotype was Ryk-specific (GFP: 606.9 ± 48.14 μm; shRyk: 893.0 ± 60.66 μm; shScr: 632.3 ± 38.53 μm; shRyk + FL-Ryk: 600.3 ± 39.99 μm, n = 6). In addition, we observed a significant increase in the total number of dendrites following Ryk depletion due to an expansion in the number of secondary and higher (2°/higher) order dendrites (Fig. 2b,d) (GFP: 8.83 ± 0.54; shRyk: 17.17 ± 1.80; shScr: 12.00 ± 1.77; shRyk + FL-Ryk: 11.33 ± 1.38; n = 6). The number of primary dendrites was not affected by the loss of Ryk. Down-regulation of Ryk using miRyk also resulted in an increase in both dendrite length and the number of 2°/higher order dendrites (Supplementary Fig. 1e,f) (length: GFP: 606.9 ± 48.82 μm; miRyk: 751.6 ± 41.06 μm; miCo: 512.6 ± 31.84 μm; miRyk + FL-Ryk: 549.4 ± 34.51 μm; 2°/higher dendrites: GFP: 7.41 ± 0.76; miRyk: 14.22 ± 2.09; miCo: 9.15 ± 1.17; miRyk + FL-Ryk: 9.86 ± 0.94; n = 6). Cotransfection of miRNA-resistant Ryk with miRyk fully rescued dendritic growth and branching, demonstrating the specificity of the miRyk sequence. Therefore, depletion of Ryk in cultured hippocampal neurons promoted dendritic growth and branching, suggesting that Ryk is a negative regulator of dendrite morphogenesis.

To confirm that Ryk acts to reduce dendritic complexity, we next expressed FL-Ryk (Myc-tagged) in hippocampal neurons. Myc immunolabelling performed at DIV5 showed that Ryk was concentrated in actin-rich filopodia at the tip of the extending MAP2-positive dendritic shaft. FL-Ryk was therefore appropriately positioned to influence dendrite morphogenesis (Fig. 2e). As predicted, expression of FL-Ryk led to a reduction in total dendrite length and a decreased number of 2°/higher order branches compared to cells expressing GFP alone (Fig. 2f–h) (length: GFP: 900.3 ± 46.77 μm; FL-Ryk: 709.3 ± 32.49 μm; 2°/higher order dendrites: GFP: 19.00 ± 1.80; FL-Ryk: 12.5 ± 1.38; n = 6). Together, these data confirm that Ryk inhibits dendritic branching and growth in Ctip2-positive hippocampal neurons.

### Ryk inhibits dendrite morphogenesis via its juxtamembrane domain

**Figure 3:**
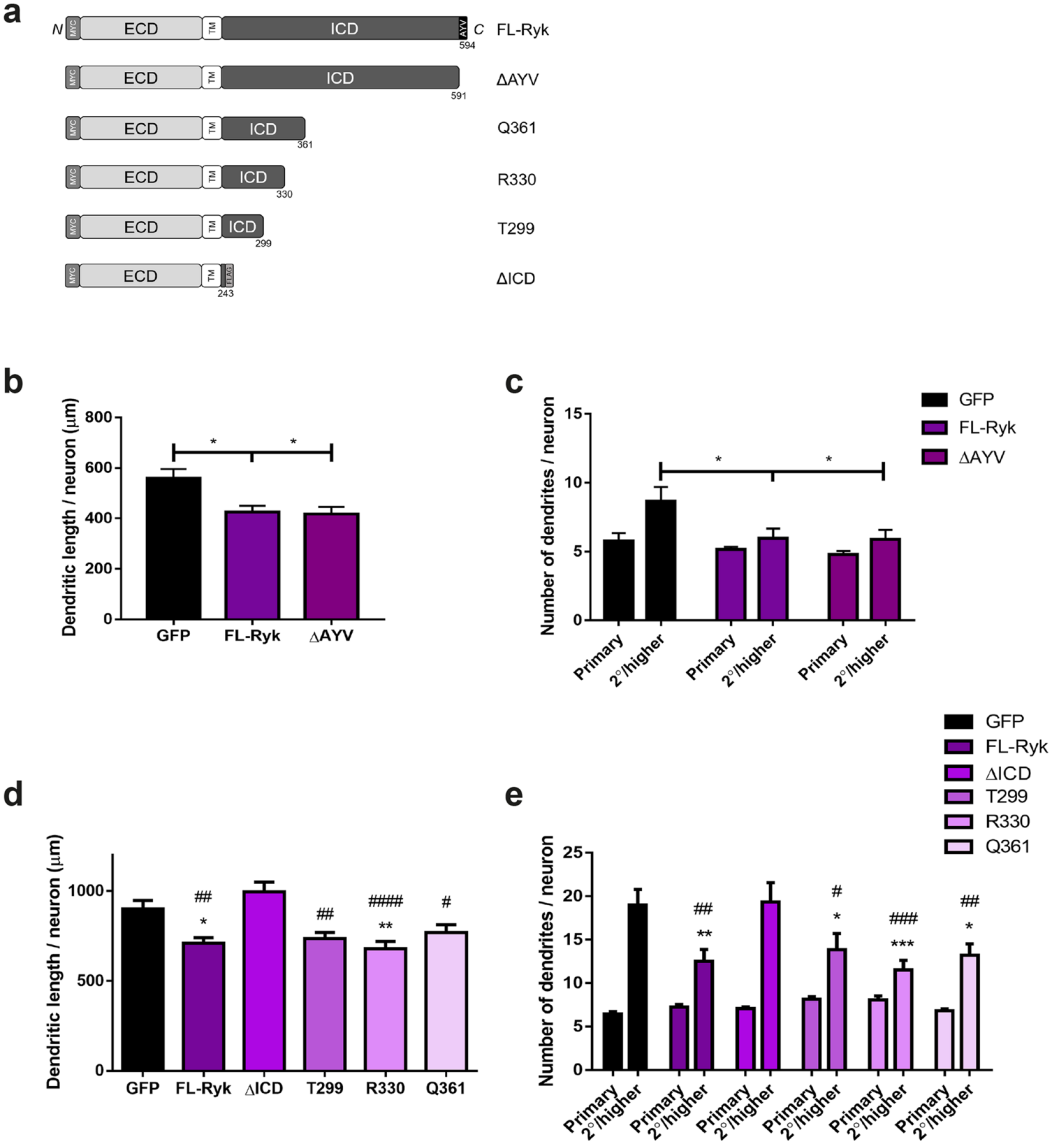
Ryk inhibits dendrite morphogenesis via its juxtamembrane domain. (**a**) Schematic diagram of FL-Ryk and truncation mutants. (**b**,**c**) Deletion of the Ryk PDZ-binding domain (ΔAYV) inhibited dendrite growth and 2°/higher order branching to the same extent as FL-Ryk (n = 6; 50–60 neurons/condition; *p < 0.05). (**d**,**e**) Similarly, the truncation mutants T299, R330 and Q361 inhibited dendrite growth and 2°/higher order branching to the same extent as FL-Ryk (n = 6; 50–60 neurons/condition; *p < 0.05; **p < 0.01; ***p < 0.001 compared to GFP alone). However, removal of the entire ICD (ΔICD) abrogated the ability of Ryk to suppress dendrite growth and 2°/higher order branching (^#^ *p* < 0.05; ^##^ *p* < 0.01; ^###^ *p* < 0.001; ^####^ *p* < 0.0001). Data are represented as the mean ± s.e.m. ECD, extracellular domain; ICD, intracellular domain; TM, transmembrane domain.

Ryk has been shown to bind the Wnt effectors Dvl and Vangl2 via its C-terminal PDZ-binding domain^23, 32, 33^. To determine whether Ryk regulates dendritic complexity through its interaction with these proteins, we removed the PDZ-binding domain (ie. the C-terminal residues AYV; RykΔAYV; Fig. 3a). Expression of RykΔAYV in DIV3 hippocampal neurons induced a significant decrease in both dendritic growth and 2°/higher order branching compared to neurons expressing GFP alone (Fig. 3b,c). This reduction in dendritic branching was equivalent to that observed for FL-Ryk (length: GFP: 558.1 ± 38.26 μm; FL-Ryk: 424.7 ± 25.87 μm; RykΔAYV: 417.7 ± 28.26 μm; 2°/higher order dendrites: GFP: 8.65 ± 1.04; FL-Ryk: 5.97 ± 0.71; RykΔAYV: 5.90 ± 0.68; n = 6), Therefore, the PDZ-binding domain is not essential for Ryk inhibition of dendrite morphogenesis. As all Wnt signalling pathways are reliant on the downstream effector Dvl^9, 10, 24, 25^, these data provide evidence that Ryk uses an alternative signalling pathway to restrict dendrite complexity.

To identify the region within the Ryk intracellular domain (ICD) responsible for the suppression of dendritic arborisation, we generated a series of truncation mutants (Fig. 3a) and assessed their effect on dendrite growth and branching. Immunolabelling of hippocampal neurons expressing these mutants revealed that, as for FL-Ryk, all mutants localized to the dendritic filopodia (Supplementary Fig. 3a), indicating that targeting of Ryk in the growing dendrite was not dependent on its cytoplasmic domain. As shown above, expression of FL-Ryk significantly reduced dendritic complexity compared to the GFP controls (Fig. 3d,e). However, removal of the entire ICD (RykΔICD) abrogated this suppressive activity as both dendritic growth and 2°/higher order branching were equivalent to those of control cells (Fig. 3d,e), demonstrating that the ICD is required for Ryk function. Note that RykΔICD was found on the plasma membrane, indicating that its cell surface expression was not affected (Supplementary Fig. 3b).

In contrast to RykΔICD, mutants in which the ICD had been truncated at amino acid Q361, R330 or T299 fully retained their ability to suppress the expansion of the dendritic tree (Fig. 3d,e). In these experiments we observed a significant reduction in growth and 2°/higher order branching after transfection of each mutant compared to GFP alone. This decrease in growth and branching was equivalent to that observed for FL-Ryk. These results identify a small juxtamembrane region of the Ryk ICD as essential for Ryk-mediated suppression of dendrite morphogenesis. As RykΔICD terminates 56 amino acids N-terminal to the T299 truncation (ie. at amino acid 243), the core region involved in regulating dendrite complexity lies within the short region between amino acids 243 and 299 (56 amino acids).

### Ryk restricts dendritic complexity in cortical neurons both *in vitro* and *in vivo*

**Figure 4:**
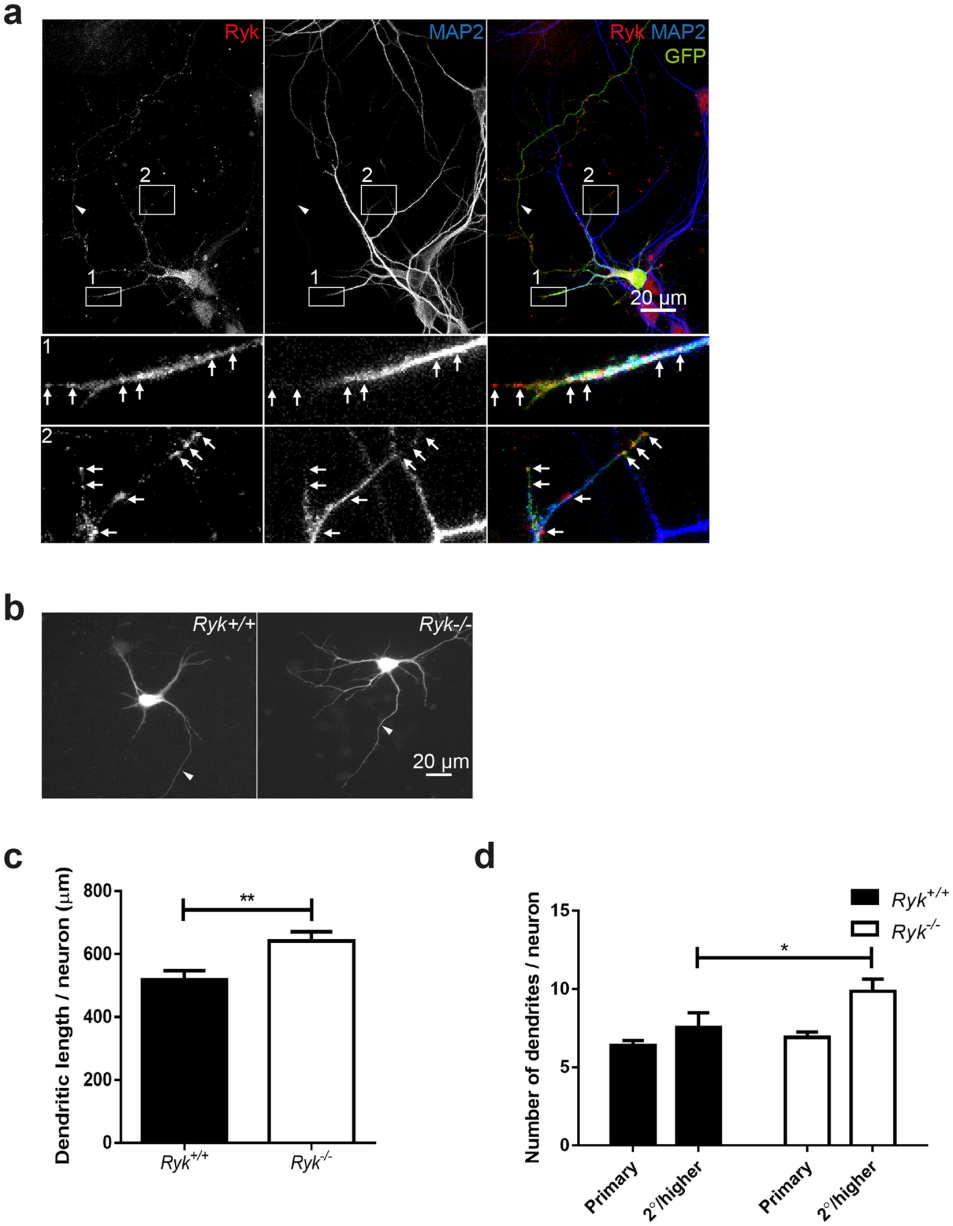
Ryk suppresses cortical dendrite complexity *in vitro*. (**a**) When transfected into wildtype cortical neurons, FL-Ryk accumulated in puncta along the dendritic shaft and was also localized to dendritic filopodia. Dendrites were identified using anti-MAP2. Bottom panels 1 and 2 are enlargements of insets. Arrows point to FL-Ryk clusters. (**b**,**c**,**d**) Dendrite morphogenesis was investigated in DIV5 cultures of cortical neurons isolated from E18.5 *Ryk* ^−/−^ and *Ryk* ^+/+^ cortices. There was a significant increase in dendritic length (**b**,**c**) and the number of 2°/higher order branches (**b**,**d**) in *Ryk* ^−/−^ compared to *Ryk* ^+/+^ neurons (6 embryos/genotype; 10 neurons/embryo; *p = 0.0480; **p = 0.0039). (**a**,**b**) Arrowheads indicate the axon.

Our *in situ* analysis demonstrated that *Ryk* was expressed in all cortical layers during the time when neurons are elaborating complex dendritic structures (P3–P5, Fig. 1). Transfection of FL-Ryk into cultured cortical neurons revealed that Ryk accumulated in puncta along the dendritic shaft and was also localized to the dendritic filopodia (DIV5, Fig. 4a), again suggesting a role in dendrite morphogenesis. To determine whether Ryk was able to inhibit cortical dendrite growth and branching, we compared cortical neuronal cultures from E18.5 *Ryk* loss-of-function (*Ryk* ^−/−^) embryos and wildtype (*Ryk* ^+/+^) littermates^18, 34^. Analysis of dendrite structure at DIV5 revealed that dendritic growth and 2°/higher order branching were significantly increased in *Ryk* ^−/−^ cortical neurons, whereas the number of primary dendrites was not affected (Fig. 4b–d) (length: *Ryk* ^+/+^: 518.6 ± 29.02 μm; *Ryk* ^−/−^: 641.7 ± 29.62 μm; 2°/higher dendrites: *Ryk* ^+/+^: 7.53 ± 0.96; *Ryk* ^−/−^: 9.85 ± 0.78; n = 6). Therefore, Ryk negatively regulates dendrite complexity in cortical neurons.

**Figure 5:**
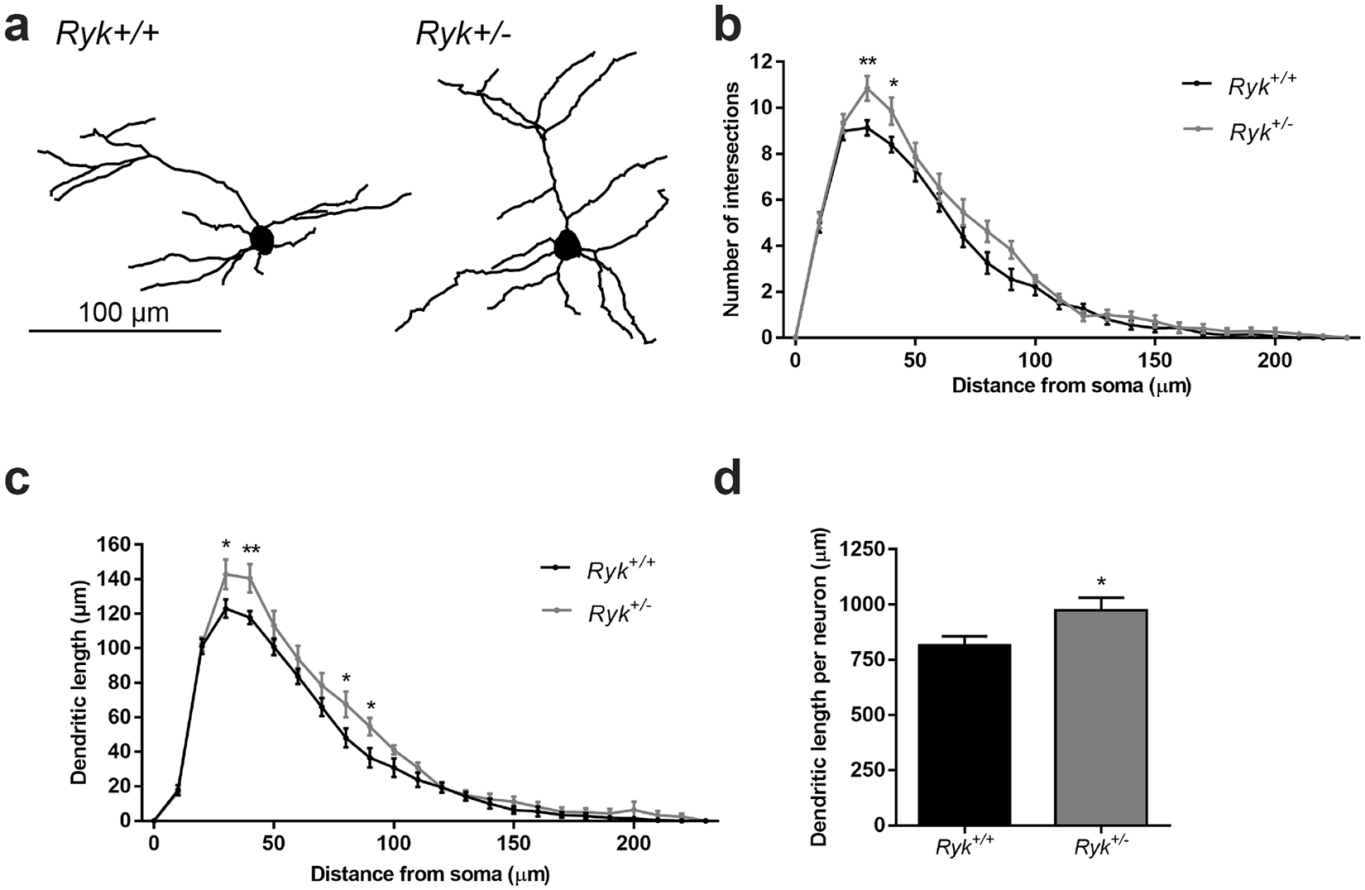
Ryk constrains layer 2/3 dendrite complexity in the somatosensory cortex. (**a**) Reconstruction of layer 2/3 pyramidal neurons in the somatosensory cortex of *Ryk* ^+/+^ (left) and *Ryk* ^+/−^ (right) P14 pups. (**b**,**c**,**d**) Sholl analysis of layer 2/3 neurons in the P14 *Ryk* ^+/−^ and *Ryk* ^+/+^ somatosensory cortex. (**b**) Compared to *Ryk* ^+/+^ neurons, there was a significant increase in the number of branches (number of intersections) within 50 μm of the soma and a trend towards increased branching at greater distances in *Ryk* ^+/−^ neurons (13 *Ryk* ^+/+^ and 12 *Ryk* ^+/−^ animals; 4 neurons/animal; *p < 0.05; **p < 0.01). (**c**) The aggregate dendrite length within each concentric radial segment was greater for *Ryk* ^+/−^ than for *Ryk* ^+/+^ neurons (*p < 0.05; **p < 0.01). (**d**) The mean total length of dendrites/neuron was greater for *Ryk* ^+/−^ neurons compared to *Ryk* ^+/+^ neurons (*p = 0.0298). Data are represented as the mean ± s.e.m.

We next investigated if loss of Ryk also increased dendritic complexity in the postnatal mouse cortex. *Ryk* is highly expressed in layer 2/3 when dendritogenesis is at its peak (Fig. 1). We therefore focused on the dendrites of layer 2/3 pyramidal neurons in the somatosensory cortex. As *Ryk* ^−/−^ embryos die prior to birth, we performed Sholl analysis on Golgi-impregnated P14 cortex from pups heterozygous for the *Ryk* loss-of-function allele. This analysis revealed a small but significant enhancement in the complexity of the dendritic tree in Ryk-depleted (*Ryk* ^+/−^) neurons. Specifically, we observed a significant increase in the number of branches (ie. number of intersections) within 50 μm of the soma and a trend towards increased branching at greater distances in the *Ryk* ^+/−^ cortex (Fig. 5a,b). In addition, the aggregate dendrite length within each concentric radial segment (Fig. 5c) and the mean total length of *Ryk* ^+/−^ dendrites per neuron (Fig. 5d) were greater for *Ryk* ^+/−^ neurons than *Ryk* ^+/+^ neurons. Together these data demonstrate that *Ryk* haploinsufficiency leads to enhanced layer 2/3 dendrite growth and branching, thereby confirming that Ryk constrains dendritic complexity in the postnatal cortex.

## Discussion

The contribution of a given neuron to the information flow within the neural circuit is governed by the structure of its dendritic arbour. The size and geometry of the dendritic tree directly determines synaptic density, type of synaptic input and position relative to the cell body, all of which influence the firing pattern of the neuron^35^. It is now clear that abnormal dendritic structure diminishes synaptic connectivity, leading to impaired cognitive function associated with conditions such as autism and schizophrenia as well as neurodegenerative disorders^1, 4, 5^. During the developmental period, exuberant extension and retraction of dendritic branches allow the neuron to explore potential territories^1, 3, 35^. To generate the unique stereotypic architecture of a given neuronal subtype, dendrite growth and branching must be constrained within predetermined boundaries. Here we identify the Wnt receptor Ryk as a novel negative regulator of dendritic arborisation and show that it is expressed at high levels in layer 2/3 pyramidal neurons of the postnatal somatosensory cortex, the granular cells of the dentate gyrus and the principal neurons within the CA1 region of the hippocampus during the postnatal period when dendritic structure is being established (Fig. 1). We demonstrate that acute loss of Ryk in actively growing hippocampal and cortical neurons promotes excessive dendrite growth and branching *in vitro*. Conversely, overexpression of wildtype Ryk suppresses these processes, confirming that Ryk acts to restrain dendrite arborisation. Notably, manipulation of Ryk activity impacted on secondary and higher order branching only, indicating that it plays a precise role in shaping the dendritic tree. Importantly, our results also provide evidence that Ryk function is conserved *in vivo* where layer 2/3 pyramidal neurons in the somatosensory cortex of *Ryk* haploinsufficient P14 animals exhibit excessive dendrite growth and branching. Therefore our study has unveiled an essential role for Ryk in sculpting dendritic structure.

Unexpectedly, in contrast to the increased dendrite arborisation seen in *Ryk* ^−/−^ cortical neurons and shRNA-depleted hippocampal neurons, a small but significant decrease in dendrite growth and branching was observed in *Ryk* ^−/−^ hippocampal neurons (Supplementary Fig. 2). This suggests that a compensatory mechanism was operating in response to genetic ablation in the *Ryk* ^−/−^ hippocampal neurons but not after acute shRNA depletion. That this compensatory mechanism was not invoked in *Ryk* ^−/−^ cortical neurons indicates that Ryk signalling plays a pivotal role in cortical dendritogenesis but may be less important for the establishment of dendrite morphology in the hippocampus.

Wnt signalling has been shown to both positively and negatively regulate dendrite morphogenesis^11, 15, 36^. However, the identity of the Wnt receptors involved and their downstream effectors are poorly characterized. The PCP pathway increases hippocampal dendrite growth and branching by prompting actin remodelling through the activation of the Rac GTPase, whereas loss of the PCP effectors Dvl1 or Vangl2 reduces dendrite complexity^11^. Conversely, removal of the C-terminal domain of Vangl2 enhances the expansion of the dendritic tree^36^, suggesting that a complex regulatory loop determines the outcome of PCP pathway activation in the dendrite. This is exemplified in the navigating axon where both Vangl2 and Dvl2, a second member of the Dishevelled family, antagonize Dvl1 in high Wnt concentrations^37–39^. Thus activation or inhibition of the PCP pathway is governed by the relative concentration of its downstream effectors. Our results demonstrate that depletion of Ryk expands the dendritic tree (Figs 2, 4 and 5), suggesting that it may antagonize the PCP pathway. Ryk is known to signal through the PCP pathway and is able to bind both Dvl and Vangl2 via its C-terminal PDZ-binding domain^23, 32, 33^. Moreover, Ryk accumulates in dendrite filopodia (Figs 2e and 4a) where both Dvl and Vangl2 are concentrated. Therefore, we first tested the hypothesis that Ryk inhibits dendrite morphogenesis through its interaction with Dvl or Vangl2. Surprisingly, we found that the Ryk truncation mutants retained full suppressive activity despite the removal of the PDZ-binding domain. This strongly argues that Ryk does not directly interact with Dvl or Vangl2 to inhibit PCP signalling. Alternatively, Ryk may suppress the PCP pathway by modulating Fzd activity. During axon navigation, Wnt/Fzd use the PCP pathway to promote attraction, whereas Ryk, acting as a Fzd coreceptor, converts Fzd-mediated attraction to repulsion via the Wnt/Ca^2+^ pathway^17, 19^. This raises the possibility that Ryk may suppress PCP pathway activity and hence the expansion of the dendritic tree by forming a complex with Fzd.

Our analysis of Ryk truncation mutants uncovered a membrane-proximal 56 amino acid region as a core subdomain that is crucial for the regulation of dendrite morphogenesis. We found that the ability of the RykT299 truncation mutant to supress dendritic arborisation was equivalent to that of full-length Ryk. In contrast, removal of the full ICD abrogated this inhibitory activity despite localization of the ΔICD mutant to dendritic filopodia (Supplementary Fig. 3a,b). This differential response places the effector domain between amino acids 243 and 299 and adjacent to the transmembrane domain. To date, interrogation of this sequence has failed to identify known protein-protein interaction motifs, and very few cytoplasmic interaction partners have been identified for Ryk. However, a recent mass spectrometry-based affinity screen has provided clues to the identity of candidate Ryk effectors that may interact with the 56 amino acid core subdomain^26^. Over 60 Ryk ICD binding partners were isolated in this screen, including the PCP components CELSR, FAT and DCHS. Interactions between Ryk and any of these proteins could negatively impact PCP signal transduction. In addition, several actin-remodelling proteins were identified, suggesting that Ryk may directly modify the actin cytoskeleton.

The ability of Ryk to inhibit dendrite growth and branching closely parallels its function as an inhibitor of axon regeneration^20, 40–42^. Wnt/Ryk interactions impede corticospinal axonal regeneration after spinal cord injury by promoting axonal retraction^20, 42^. As seen for dendrites, silencing Ryk activity promotes axonal regrowth and sprouting of collateral branches after spinal cord hemisection^20, 43^. Therefore, delineating the downstream effectors of Ryk may uncover viable targets for the design of novel therapeutic strategies to enhance axonal repair after spinal cord injury.

Within the somatosensory cortex the architecture of the dendritic arbour dictates the size of a neuron’s receptive field and is therefore the primary determinant of the sensory stimulus impinging upon the cell^3^. Here we show for the first time that Ryk acts to constrain dendrite arborisation in layer 2/3 pyramidal neurons of the somatosensory cortex. We therefore postulate that Ryk plays a central role in the establishment of the receptive field of these neurons. Although the identity of the Wnts responsible for activating Ryk is currently unknown, the primary Ryk ligand, Wnt5a, is expressed throughout the P7 cortex, including in layer 2/3 neurons^44^. Therefore, Wnt5a may trigger Ryk-mediated inhibition of dendrite growth and branching when dendritic fields are being established. This notion is supported by the finding that Wnt5a/Ryk interactions in *Drosophila* also limit dendrite growth in order to confine dendritic territories^27^, indicating that the ability of Ryk to restrict the expansion of the dendritic tree is evolutionarily conserved. Loss of Ryk in the primary motor cortex of the mouse has recently been shown to enhance the expansion of motor maps into adjacent cortical areas affected by spinal cord injury^43^. This raises the intriguing possibility that Ryk may influence the plasticity of neural circuits by modifying dendritic structure.

## Materials and Methods

### Animals

All experiments involving animals were approved by the Anatomical Biosciences Animal Ethics Committee of The University of Queensland and performed in accordance with the Australian Code of Practice for the Care and Use of Animals for Scientific Purposes. C57Bl/6 pregnant dams were obtained from The University of Queensland breeding facility. *Ryk* loss-of-function mice (C57Bl/6j × 129/Sv) were kindly provided by Prof. Steven Stacker (Peter MacCallum Cancer Centre, Melbourne, Australia)^34^. Genotyping was performed as previously described^34^. For timed-matings, males and females were placed together overnight, with the following morning being designated as E0.5.

### *In situ* hybridization

#### RNA probes

cDNAs were prepared from C57Bl/6 embryonic cortex mRNA using the SuperScript III First-Strand Synthesis System (Invitrogen) according to the manufacturer’s instructions. Gene-specific cDNA amplicons incorporating the T7 forward and SP6 reverse primer sequences were then generated by PCR using the iProof High-Fidelity PCR Polymerase Kit (Bio-Rad). Subsequently, 200 ng of amplicon was used as the template for digoxigenin (DIG)-labelled RNA probe synthesis with either T7 or SP6 RNA polymerase (Roche). Probes were purified by agarose gel electrophoresis followed by purification using the Wizard SV Gel and PCR Clean-Up Kit (Promega).

#### *In situ* hybridization

*In situ* hybridization was performed as previously described^45^. Briefly, paraffin-embedded sections were dewaxed and rehydrated in PBS, post-fixed with 4% paraformaldehyde (PFA) for 10 min, permeabilized with proteinase K (10 μg/ml for 15 min, Roche), re-fixed in 4% PFA for 5 minutes and treated with 0.25% acetic anhydride for 10 min prior to hybridization with DIG-labelled probes (1:100 in hybridization buffer; Amresco) overnight at 65 °C. Sections were washed twice at 65 °C for 30 min (1 × SSC, 50% formamide, 0.1% Tween 20) followed by treatment with 20 μg/ml RNase A (Roche) for 30 min at 37 °C. Slides were then incubated with the anti-DIG antibody (1:2500, Roche) overnight at 4 °C. Probes were detected using NBT/BCIP (Promega) according to the manufacturer’s instructions. Sections were counterstained with Nuclear Fast Red (Sigma-Aldrich), dehydrated and cleared in xylene and mounted in DPX mounting medium (Ajax Finechem). Slides were imaged using a Mirax digital slide scanner (Carl Zeiss) with a 20X objective.

### Constructs and shRNAs

Full-length Ryk (FL-Ryk) tagged at its N-terminus with two consecutive myc epitopes (EQKLISEEDL), a gift from Prof. Steven Stacker, was subcloned into pCAGIG^46^. Ryk (NCBI: NM_013649) truncation mutants were also cloned into pCAGIG. shRyk and shScr shRNAs were cloned into ﻿pCA-ß-EGFPm5-Silencer 3, a gift from Assoc. Prof. Julian Heng (Harry Perkins Institute of Medical Research, Perth, Australia). miRyk and miCo long-hairpin miRNAs^31^ were cloned into pcDNA6.2-GW-EmGFP-miR^47^ using the BLOCK-iT Pol II miR RNAi Expression Vector Kit (Invitrogen). The EmGFP-miR cassettes for each sequence were then subcloned into the piggyBac vector, a gift from Prof. Joseph LoTurco (University of Connecticut, USA)^48^, replacing the GFP sequence. The sequences were as follows: shRyk: GAAAGATGGTTACCGAATA, shScr: GGGTCCAATCGATAATAGGA, miRyk: GCAGCTCAATCTGACAGTGAA, miCo: AAATGTACTGCGCGTGGAGAC. To generate RNAi-resistant FL-Ryk cDNAs nucleotide changes in the shRyk or miRyk binding site (shRyk-resistant FL-Ryk A1765G and T1771G; miRyk-resistant FL-Ryk C607T and G613T, respectively; NCBI NM_013649) were introduced using the QuickChange XL Site-Directed Mutagenesis Kit (Agilent Technologies).

### Primary neuronal culture and transfections

Dissociated hippocampal and cortical neuronal cultures were prepared from E18.5 mouse embryos as described previously^49^. Briefly, the tissue was trypsinized for 20 min, dissociated by pipetting, and seeded at a concentration of 7.5 × 10^4^ hippocampal neurons/well or 1 × 10^5^ cortical neurons/well on glass coverslips (12 mm diameter) coated with poly-L-lysine (Sigma-Aldrich) and laminin (Gibco, Invitrogen). The neurons were cultured for 5 days *in vitro* in Neurobasal medium (Gibco) supplemented with 2% B27 (Gibco), 0.25% L-glutamine (Gibco), penicillin (10 U/ml) and streptomycin (10 μg/ml) (Gibco). For hippocampal cultures, neurons from all littermates were pooled before plating. For *Ryk* ^−/−^ and *Ryk* ^+/+^ cortical cultures, neurons derived from individual embryos were plated separately. Neurons were transfected at DIV3 with shRNAs (1.5 μg), miRNAs (1.5 μg) or Ryk cDNAs (1.0 μg) using Lipofectamine 2000 (Invitrogen) according to the manufacturer’s instructions and PFA-fixed at DIV5.

### Immunocytochemistry and antibodies

Neuronal cultures were fixed with 4% PFA. Coverslips were then rinsed × 3 in PBS and incubated at room temperature for 1 h in PBS, 0.2% Triton X-100, 4% donkey serum (Sigma-Aldrich), followed by primary antibody (1 h, PBS, 0.2% Triton X-100) and then secondary antibody before mounting in ProlongGold Antifade Reagent (Molecular Probes). Primary antibodies: mouse anti-Myc (1:500; Sigma-Aldrich, #M4439, clone number 9E10), rabbit anti-Myc (1:500; Millipore, #06–549), rat anti-Ctip2 (1:500; Abcam, #ab18465), rabbit anti-MAP2 (1:500; Millipore, #AB5622), goat anti-GFP-FITC (1:1000; Abcam, #ab6662). Secondary antibodies were conjugated to Alexa Fluor 488, 546, 568, or 647 (1:1000; Molecular Probes). Images were acquired on a Diskovery spinning disk microscope with a 60X objective, numerical aperture: 1.4 (Nikon), with a z-step of 130 nm (xy pixel size: 93 nm) and deconvolved using Huygens Professional v16.10 software (Scientific Volume Imaging).

### Quantification of dendrite length and branching

Images were acquired on a Zeiss AxioImager microscope with a 20X objective, numerical aperture: 0.5 and customized filter sets. Cultured neurons with equivalent GFP expression were chosen for quantitative analyses of dendritic length and branch number using the NeuronJ plugin of ImageJ. All dendrites were semi-manually traced and labelled as primary (dendrite originating from the soma), secondary (extending from the primary dendrite) or higher-order dendrites. Filopodia shorter than 10 μm were excluded from the analysis. Statistical analysis was performed using GraphPad Prism (version 7, GraphPad Software Inc.). Gaussian distribution was assessed using the D’Agostino and Pearson normality test. An unpaired Student’s *t*-test was used for normally distributed data from two groups. A Mann-Whitney test was used for non-Gaussian data. For more than two groups, Gaussian data were analysed using one-way ANOVA followed by Tukey’s *post hoc* test. For non-Gaussian data a Kruskal-Wallis test was used followed by a Dunn’s *post hoc* test. Finally, dendrite type was analysed using 2-way ANOVA followed by a Tukey’s *post hoc* test. Statistical significance was considered to be *p* < 0.05. The sample size was calculated for an alpha of 0.05 and a power of 0.8.

### Golgi staining and Sholl analysis

Thick vibratome sections (coronal plane, 150 μm) were cut from the brains of P14 *Ryk* ^+/−^ and *Ryk* ^+/+^ littermates and Golgi stained using a sliceGolgi Kit (Bioenno) according to the manufacturer’s protocol. Briefly, brains were fixed for 5 h at room temperature in the sliceGolgi Kit fixative and then rinsed in 0.1 M phosphate buffer (20 mM NaH_2_PO_4_ **·**H_2_O, 80 mM Na_2_HPO_4_ **·**7H_2_O) for 2 h at room temperature. Sections were then incubated in the dark in impregnation solution for 5 days at room temperature. Finally, sections were stained for 5 min in staining solution and post-stained for 3 min before mounting on gelatin-coated Superfrost microscope slides (Menzel-Gläser).

Brightfield images were taken with a 20X objective on a Zeiss AxioImager in three dimensions with a z-step of 0.5 μm. Fully labelled neurons located within layer 2/3 of the somatosensory cortex were traced using the user-guided dendrite tracing tools of Neurolucida 360 software (MBF Bioscience). Sholl analysis was performed in Neurolucida Explorer using a radius of 10 µm. Statistical analysis was performed in GraphPad Prism using 2-way ANOVA followed by Sidak’s multiple comparison test. For total length, the Gaussian distribution was determined with the D’Agostino and Pearson Normality test and statistical significance was determined using the Mann-Whitney test. Statistical significance was considered to be *p* < 0.05. The sample size was calculated for an alpha of 0.05 and a power of 0.8.

## Electronic supplementary material

Supplementary information
