# Supplementary material for: The Wnt receptor Ryk is a negative regulator of mammalian dendrite morphogenesis
Source: Sci Rep. 2017 Jul 20;7:5965. doi: 10.1038/s41598-017-06140-z (PMC5519545; doi:10.1038/s41598-017-06140-z)

The Wnt receptor Ryk is a negative regulator of mammalian dendrite morphogenesis

Vanessa Lanoue, Michael Langford, Amanda White, Kai Sempert, Lily Fogg, and Helen M. Cooper*

The University of Queensland, Queensland Brain Institute, Brisbane, Queensland, 4072, Australia.

SUPPLEMENTARY FIGURE LEGENDS

Supplementary Figure 1 Validation of RNAi constructs. (**a**,**b**) Validation of Ryk RNAi knockdown in HEK293T cells showed a reduction of 83% or 84% in FL-Ryk protein levels relative to α-tubulin (α-Tub) after cotransfection of Myc-tagged FL-Ryk with either shRyk or miRyk, respectively. Controls: shScr, miCo. (n = 3, ***p = 0.0004; ****$p < 0.0001$, Student's *t*-test; mean ± s.e.m). (**c**,**d**) Validation of the shRNA-resistant FL-Ryk (**c**) and miRNA-resistant FL-Ryk (**d**) constructs in HEK293T cells. Western blots show that the level of RNAi-resistant FL-Ryk was not reduced in the presence of shRyk or miRyk. (**e**,**f**) Plasmids containing Ryk-specific miRNA (miRyk) or control miRNA (miCo) and GFP were transfected into DIV3 hippocampal neurons and dendritogenesis was assessed at DIV5. Depletion of Ryk resulted in an increase in dendritic length (**e**) and the number of secondary and higher order (2°/higher) dendrites (**f**). Coexpression of RNAi resistant FL-Ryk and miRyk fully rescued dendritic growth and branching. (n = 6; 50-60 neurons/condition; *$p < 0.05$; **$p < 0.01$; ***$p < 0.001$; ****$p < 0.0001$). Data are represented as the mean ± s.e.m.

Supplementary Figure 2 Dendrite growth and branching is enhanced in cultured *Ryk*$^{-/-}$ hippocampal neurons. Dendrite morphogenesis was investigated in DIV5 cultures of hippocampal neurons isolated from E18.5 *Ryk*$^{-/-}$ and *Ryk*$^{+/+}$ cortices. There was a small but significant decrease in dendritic length (**a**,**b**) and the number of 2°/higher order branches (**a**,**c**) in *Ryk*$^{-/-}$ compared to *Ryk*$^{+/+}$ neurons (4 embryos *Ryk*$^{+/+}$ and 8 embryos *Ryk*$^{-/-}$; 10 neurons/embryo; *p = 0.0290; **p = 0.0090). **a**,**b**: Arrowheads indicate the axon.

Supplementary Figure 3 The truncated Ryk proteins localize to dendritic filopodia. (**a**) Immunolabelling demonstrated that the Ryk truncation mutants were present on the dendritic shaft and concentrated in the dendritic filopodia of DIV5 hippocampal neurons. (**b**) Immunolabelling in the

absence of detergent revealed that RykΔICD and FL-Ryk were localized to the plasma membrane of transfected HEK293T cells.

Supplementary Figure 1

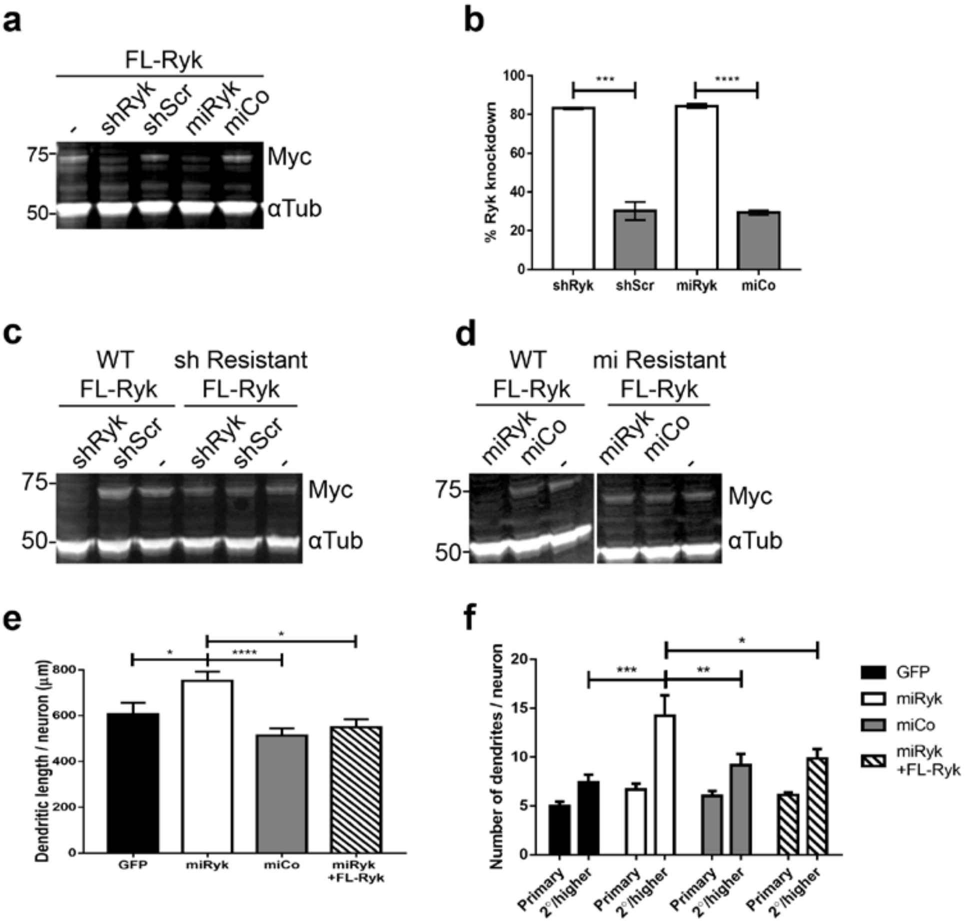

Supplementary Figure 2

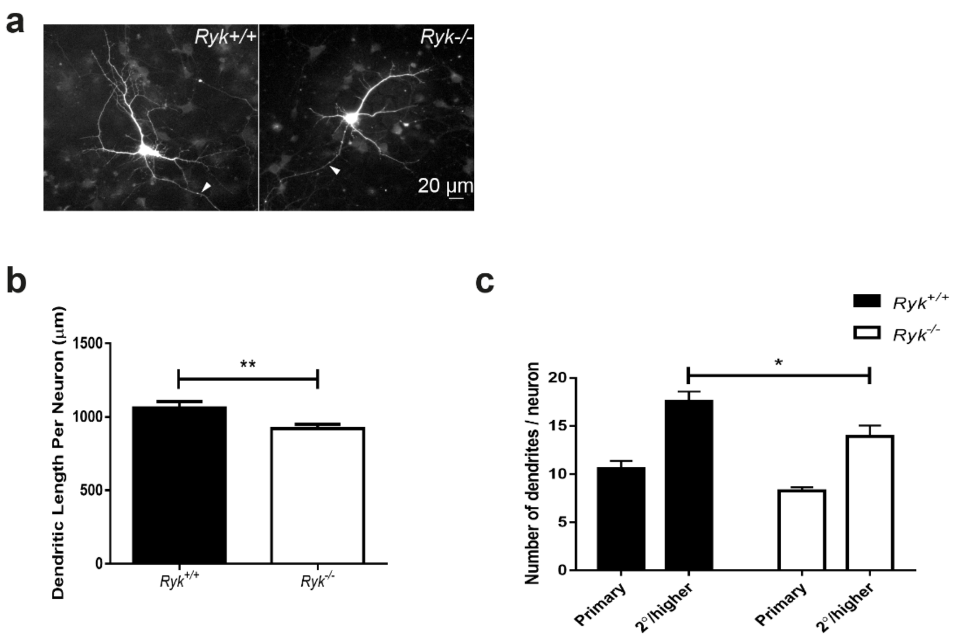

Supplementary Figure 3

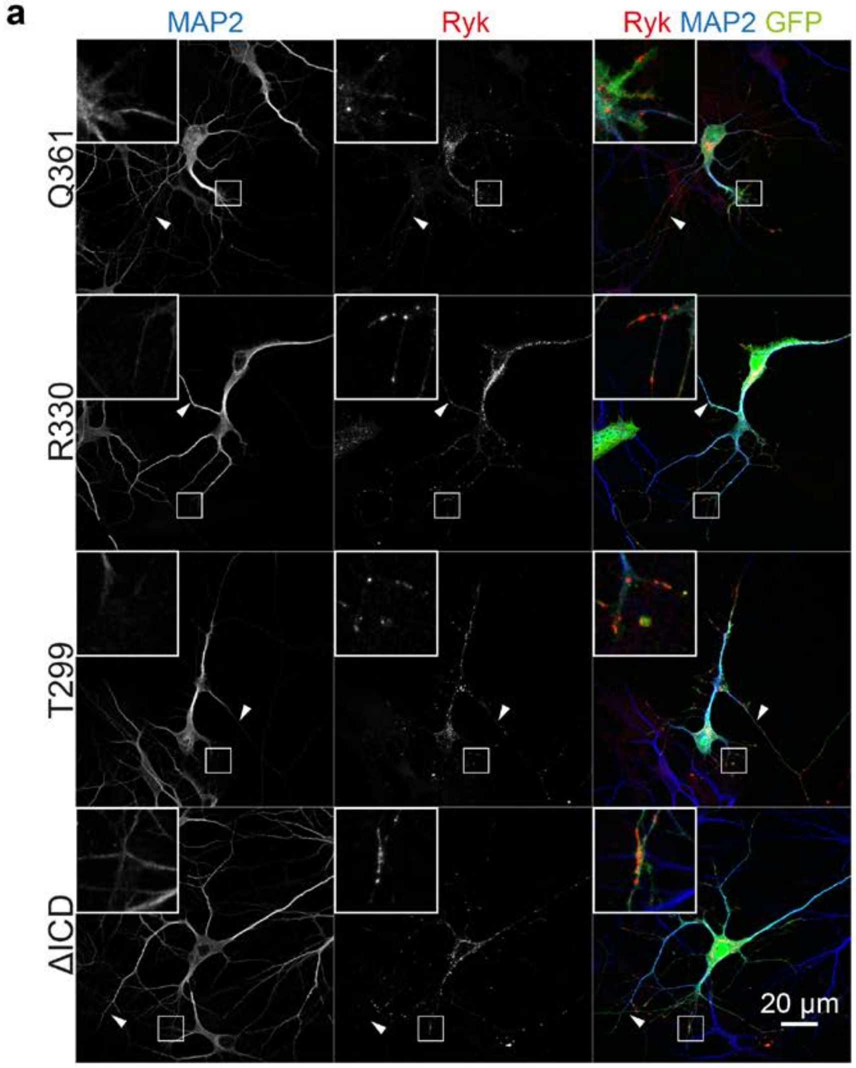

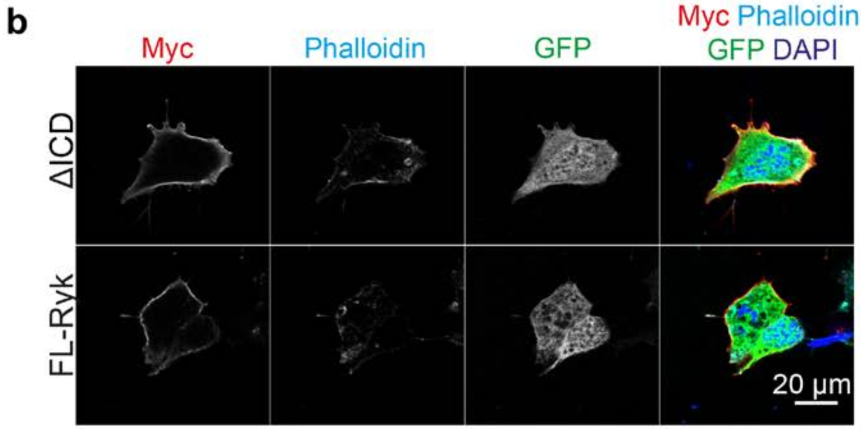

Supplement: Supplementary file 1 — Supplementary information [file 41598_2017_6140_MOESM1_ESM.pdf]
